# Flight training changes the brain functional pattern in cadets

**DOI:** 10.3389/fnins.2023.1120628

**Published:** 2023-03-21

**Authors:** Xi Chen, Zian Wang, Hao Jiang, Yu Meng, Hongmei Wang, You Li, Kaijun Xu, Jiazhong Yang, Cheng Luo

**Affiliations:** ^1^Institute of Flight Technology, Civil Aviation Flight University of China, Guanghan, China; ^2^Institute of Marxism, Civil Aviation Flight University of China, Guanghan, China; ^3^Department of Student Affairs, Civil Aviation Flight University of China, Guanghan, China; ^4^Key Laboratory for NeuroInformation of Ministry of Education, School of Life Science and Technology, University of Electronic Science and Technology of China, Chengdu, China

**Keywords:** flying cadets, resting-state fMRI, degree centrality, flying training, executive function

## Abstract

**Introduction:**

To our knowledge, this is the first study to use MRI (Magnetic Resonance Imaging) before and after an intensive flight training. This study aimed to investigate the effectiveness of flight training in civil flying cadets.

**Methods:**

The civil flying cadets and controls completed two study visits. Visit 1 was performed in 2019, and high spatial resolution structural image and resting-state functional MRI data were collected. The second visit was completed in 2022. In addition to the MRI data mentioned above, participants completed the cognitive function assessment at the second visit.

**Results:**

Mixed-effect regression model analysis found that flight training enhanced the degree centrality (DC) values of the left middle frontal gyrus and left lingual gyrus. The subsequent correlation calculation analysis suggested a possible relationship between these alterations and cognitive function.

**Discussion:**

These results suggest that flight training might promote the DC value of the prefrontal and occipital cortices and, in turn, enhance their executive function.

## 1. Introduction

During World War I, flying cadet applicants had to meet rigid physical standards. Nowadays, because automated equipment is heavily implemented, flying is a mental work rather than physical work. It requires many cognitive activities rather than physical activities. Therefore, the training was not just physical. Training for cognitive abilities, such as decision-making, assumptions, reasoning, etc., is more important. Traditional international standards and national regulations for airline pilot training are largely based on evidence of accidents involving jet aircraft in the early generations. This flight training standard lasted for approximately 100 years, but the effects of training on the brain remain unknown. Previous studies have identified that cognitive training can improve cognitive functioning and change brain functional factors and brain shape characters (Lerch et al., 2011; Meyer et al., 2017; Baykara et al., 2021). Since flight training is mostly cognitive training, we hypothesized that flight training may alter the brain characteristics to some degree.

Neuroimaging is a widely used technique for investigating the human brain. Resting-state functional MRI (Magnetic Resonance Imaging) data can depict intrinsic brain functional characteristics. Our previous studies with pilots had shown that the functional MRI could depict the psychophysiological alterations of flying. The pilots exhibited better functional dynamics and cognitive flexibility (Chen et al., 2020). In the current study, we focused on the cognitive function changes in cadets. Degree centrality (DC) is a graph-based measurement of the brain’s functional characteristics. The DC value represents the node characteristics of the intrinsic brain connectivity networks. For a given voxel, Pearson correlations between the time courses of the target voxel and that of each voxel in the whole brain were calculated. This procedure was performed across the entire brain. Then, a voxel with high values suggested its central role in transferring information across the brain. This value is often used to detect changes in the brain network (Zhang et al., 2011; Sinha and Nagarajaram, 2013; Yang et al., 2014; Liu et al., 2015; Takeuchi et al., 2015; Candeloro et al., 2016). In the current study, we used DC to map the neural alterations of flight training-induced brain functional character. We collected functional brain MRI data from civil flying cadets and controls at two visits. The Berg Card-Sorting Test-64, an executive function feature-switching task, was used to assess the participants’ cognitive function. We hypothesized that intrinsic brain activity may be altered in flying cadets after flight training and that these changes are related to their cognitive functions. These findings may contribute to the mapping of the neural mechanisms of flight training.

## 2. Materials and methods

### 2.1. Study design and participants

The study protocol was approved by the Ethics Committee of the University of Electronic Science and Technology of China (Chengdu, China; No. 2019–042019).

All participants (including flying cadets and controls) were undergraduates who enrolled at the same time from the Civil Aviation Flight University of China. The participants were originally recruited through advertisements and were given compensation. Individuals who agreed to contact for future studies were included. The exclusion criteria were traumatic brain injury, substance-related disorders, and a history of neurological illness. All the participants were naïve to MRI. All participants provided written informed consent to participate in this study. Finally, 25 male flying cadets and 24 male controls were recruited. The aircraft types of cadets were C172R, SR-20 and DA-42. They all completed 40 h simulation training, and the actual flight training time could be seen in Table 1. The controls were majoring in Psychology or Aeronautical Engineering and had no experience with flying and flight simulator. The two groups were matched for sex, handedness, and age. All the data were collected at the Center for Information in Medicine of the University of Electronic Science and Technology of China.

**Table 1:** Demographic characteristics of the two groups at the first time point.

	Flying cadets (*N* = 25)		Controls (*N* = 24)		Significance	
	M	SD	M	SD	*T*-value (Chi-square test)^*^	*p*-Value
Age (years)	18.84	0.69	18.88	0.85	−0.159^a^	0.88
Gender (% male)	100%		100%			
Education (years)	12		12			
Handedness (% right)^c^	96		92		0.40^b^	0.53
Total flight time (simulation flying) (hours)	40	0				
Total flight time (actual flying) (hours)	181.96	30.90				

M, mean value; SD, standard deviation. ^a^Two-tailed *t*-tests. ^b^Chi-square tests. ^c^The proportion of right-handed people in the total population.

* The different models of computation for continuous and discrete variables.

The participants completed two study visits. The first visit took place when they had just come to the university as freshmen in 2019. The second visit was completed in 2022. The interval between the two visits of the participants ranged from 31 to 41 months. During the visit 1, high spatial resolution structural images (T1) and resting-state functional MRI (fMRI) data were collected. In the second visit, in addition to the aforementioned MRI data, participants completed the cognitive function assessment.

### 2.2. Data acquisition

MRI data were acquired using a 3-T MRI scanner (DISCOVERY MR 750, GE Healthcare, Waukesha, WI, United States). T1 images were collected using a three-dimensional spoiled gradient echo pulse sequence with the following parameters: repetition time (TR) = 5.976 ms; echo time (TE) = 1.976 ms; flip angle = 9°; field of view (FOV) = 256 mm × 256 mm × 154 mm; matrix = 256 × 256; slice number = 154; the voxel size was 1 mm × 1 mm × 1 mm. FMRI data were collected using a gradient-echo echo-planar imaging sequence with the following parameters: TR = 2,000 ms, TE =30 ms, flip angle = 90°, FOV = 240 mm × 240 mm × 140 mm, matrix = 64 × 64, slice thickness = 4 mm (no gap). Thus, the in-plane voxel size was 3.75 mm × 3.75 mm × 4 mm. Each volume contained 35 slices. In total, 255 volumes were acquired from each participant.

Cognitive function was measured using the Psychology Experiment Building Language, which includes computerized versions of 94 psychological tests (Mueller and Piper, 2014). We used the Berg Card-Sorting Test-64 to assess the participants’ cognitive function. The “Berg Card-sorting Test-64″ is an executive function feature-switching task. This was a free and alternative implementation of the Wisconsin Card Sort-64 (Fox et al., 2013). It measures executive functioning and mental flexibility (Fox et al., 2013). The test lasted about 8 min and was conducted in a default way. During the testing process, the participants were required to sort the cards according to an unknown and changing rule. The rules changed when the number of correct response was up to 10 (the implication was the completion of a category; Heaton et al., 1993). Total accuracy, perseverative responses (PR), perseverative errors (PE), and total reaction time were collected to represent mental flexibility and executive function (Greve et al., 2005). PR and PE are two key variables to assess cognitive flexibility. PR are the persistent responses made on the basis of a previous or novel rule, regardless of the feedbacks. The incorrect PR are termed PE. In addition, the flight hours of each cadet were recorded.

### 2.3. MRI data preprocessing

MRI data preprocessing was conducted with the SPM12 (Statistical Parametric Mapping 12, http://www.fil.ion.ucl.ac.uk/spm/). The T1 images were segmented into gray matter, white matter, and cerebrospinal fluid. The first five scans of the fMRI data were removed for magnetization equilibrium. The remaining 250 scans were then subjected to slice timing and head-motion correction. Participants who exceeded head motion of 2 mm in any direction or 2° rotation in any direction were excluded from the subsequent analysis. The images were then co-registered to the structural gray matter images and normalized to the standard MNI template at 3 × 3 × 3 mm^3^ resolution. Then, the images were processed to remove the linear trends and bandpass filtered (0.01–0.08 Hz). The white matter signal, cerebrospinal fluid signal, and head motion parameters (rigid-body 6 parameters) were regressed as nuisance covariates.

### 2.4. Degree centrality analysis

Voxel-wise DC was performed using Restplus software^1^ in the whole brain. For a given voxel, Pearson correlations between the time courses of the target voxel and that of each voxel in the whole brain were calculated. The procedure was performed across the entire brain. Finally, we acquired Pearson’s correlation coefficient matrix between any pair of voxels in the brain. Positive correlations above a threshold of r = 0.25 were included in further analysis (Buckner et al., 2009). The resulting data were finally smoothed using a 6-mm full-width at half-maximum Gaussian kernel. Correlation coefficients were then transformed into normally distributed Fisher Z-scores. The weighted DC value of a given voxel was identified as the sum of the significant connections (Z values) between the voxel and all the other voxels. A voxel with high values suggested its central role in transferring information across the brain.

### 2.5. Statistical analysis

Differences in neuropsychological traits between the two groups were compared using an independent *t*-test (*p* < 0.05).

For fMRI data analysis, a mixed-effect regression model was used to compare changes in DC values between the two groups [Gaussian Random Field (GRF) correction, *p* < 0.05 at the cluster level, and with an initial height threshold of *p* < 0.001]. The degree of freedom of interaction effect was 1((2–1) *(2–1)). The interaction effect between time and group suggested the flight-training effect. The main effect of the group assessed individual differences between flying cadets and other college students. The main effect of time represented the spontaneous change in living and learning at university. In the current study, we focused on the interaction effect.

In addition, Pearson correlation was calculated to estimate the relationship between the brain functional properties resulting from the mixed-effect regression analyze and neuropsychological traits.

## 3. Results

### 3.1. Participants

No cadet was excluded because of excessive head motion. Then, there were 25 flying cadets and 24 controls included. The demographic parameters are shown in Table 1. Age, sex, handedness, and education level were not significantly different between the two groups.

### 3.2. Neuropsychological results

Neuropsychological data were not collected at visit 1. At visit 2, there were no significant differences between the groups (Table 2).

**Table 2:** Characteristics of the two groups at the second time point.

	Flying cadets (*N* = 25)		Controls (*N* = 24)		Significance	
	M	SD	M	SD	T-value	*p*-Value
Bcst_total accuracy (%)	80.87	5.38	80.47	8.51	0.20	0.84
Bcst_reaction time (ms)	2031.79	608.19	1791.59	367.62	1.68	0.10
Bcst_perseverative response	18.80	3.00	19.50	2.70	−0.86	0.40
Bcst_perseverative error	6.84	1.40	7.17	2.53	−0.56	0.58

M, mean value; SD, standard deviation.

* *p* < 0.05.

### 3.3. FMRI results

**Figure 1:**
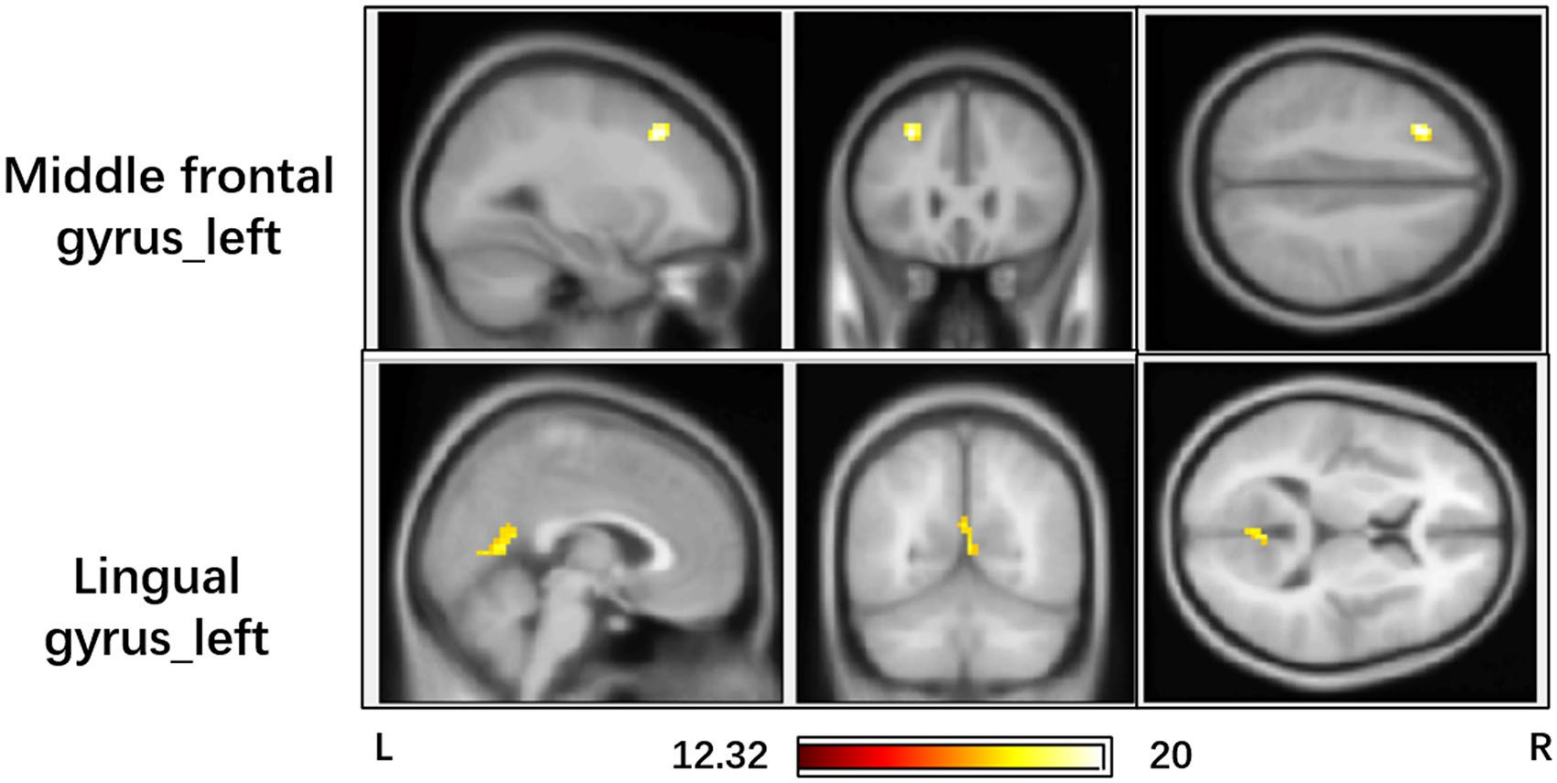
Brain regions with significant interaction effect (GRF corrected, *p* < 0.05, the initial height threshold is *p* < 0.001).

**Figure 2:**
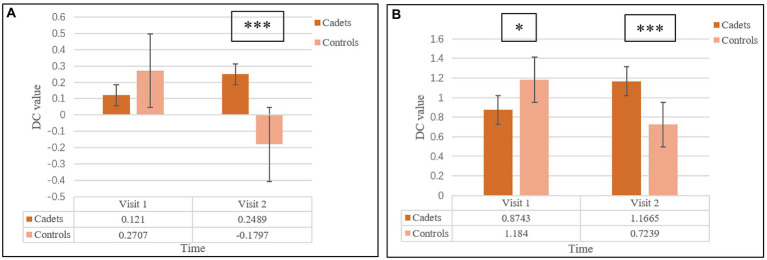
Detailed information of the interaction effect of the two groups at the two visits. **(A)** represented the detailed information of the left middle frontal gyrus; **(B)** represented the information of the lingual gyrus. One star represented the *p* < 0.05, three stars represented the *p* < 0.001.

The interaction effect (group vs. time) in the mixed-model analysis is shown in Figures 1, 2 and Table 3. This represents the difference between the changes in the two groups at the two time points. The left middle frontal gyrus and left lingual gyrus exhibited significant interaction in DC values (GRF correction, *p* < 0.05 at the cluster level, and with an initial height threshold of *p* < 0.001).

**Table 3:** Brain regions with significant interaction effect (GRF corrected, *p* < 0.05, the initial height threshold is *p* < 0.001).

	Center (MNI)			Peak *F* value	Brain regions (AAL)	Cluster size (voxels)
	x	y	z			
1	0	−66	6	16.14	Left lingual gyrus	43
2	−27	24	42	21.74	Left middle frontal gyrus	31

**Figure 3:**
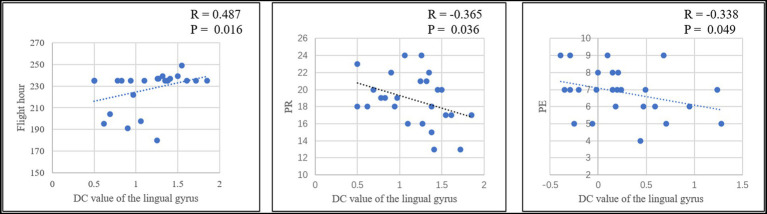
The significant relationship between the brain functional properties and behavioral indices in cadets (*p* < 0.05).

**Figure 4:**
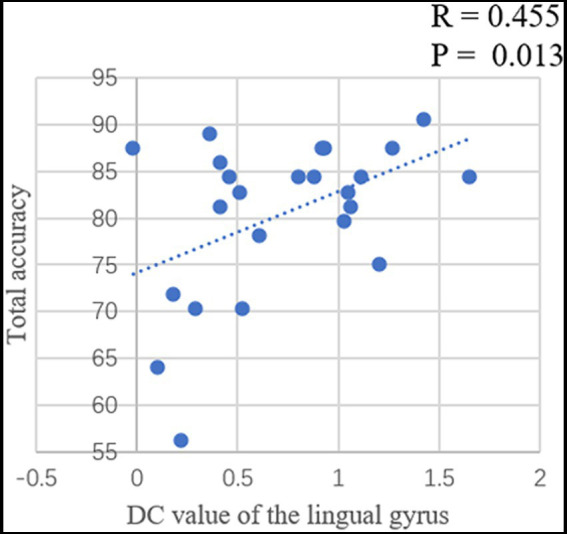
The significant relationship between the brain functional properties and behavioral indices in controls (*p* < 0.05).

**Figure 5:**
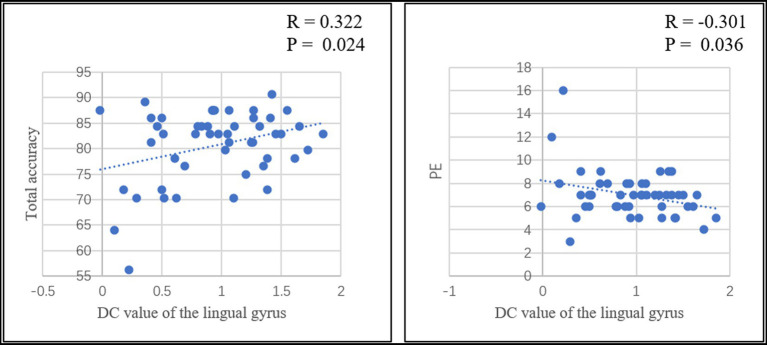
The significant relationship between the brain functional properties and behavioral indices in the combined groups of the two groups (*p* < 0.05).

Pearson correlation analysis was conducted between the DC value of left middle frontal gyrus and left lingual gyrus and total flight hours (in cadets), total accuracy, PR, PE and total reaction time of the card sorting test in each group. In cadets, a positive correlation was identified (r = 0.487, *p* = 0.016) between total flight hours and the DC value of the lingual gyrus after eliminating singular values (Figure 3). In addition, the PR value was significantly negatively correlated with the DC value of the lingual gyrus (r = −0.365, *p* = 0.036), and the PE value significantly negatively correlated with the DC value of the middle frontal gyrus (r = −0.338, *p* = 0.049; Figure 3). In controls, the total accuracy was positively correlated with the DC value of the lingual gyrus (r = 0.455, *p* = 0.013; Figure 4). In addition, when the two groups combined into one group, a positive correlation was identified (r = 0.322, *p* = 0.024) between total accuracy and the DC value of the lingual gyrus; and a negative correlation was identified between the PE value and the DC value of the lingual gyrus (r = −0.301, *p* = 0.036; Figure 5). While after multiple correlations (*p* < 0.005 in cadets; *p* < 0.006 in other groups), these associations were no longer significant. The reason for this result might be the small number of participants.

## 4. Discussion

This study investigated the effectiveness of flight training in flying cadets. Training enhanced the DC values of the left middle frontal gyrus and the left lingual gyrus. Moreover, the subsequent correlation calculation analysis suggested a possible relationship between these alterations and cognitive function.

The card sorting test is the most commonly used instrument in clinical practice to assess executive functioning, such as inhibitory processes, attentional reorientation and abstract reasoning. The stimuli printed on the cards differed in shape, color, and number variations. Participants were required to classify the cards based on these three dimensions. In addition, the correct sorting principle was changed several times, while the participants were not informed. Feedback from each trial was immediately provided to the participant throughout the experiment. Three core cognitive components were included in this test. First, when a negative reward is given, the current rule is changed rapidly; second, the rules and answers are memorized to avoid invalid tests; and third, reasoning is based on feedback (Dehaene and Changeux, 1991). Total accuracy reflects the level of executive ability. The higher the accuracy, the stronger the ability. Meanwhile, the PE and PR are the most important indices of the card sorting test. Perseveration in the card sorting test was thought to reflect failure to change strategies according to environmental demand. PR represents perseverative responses. PE is the number of incorrect responses in which the participant continues to use a previously correct rule (Grant and Berg, 1993). The higher levels of PE and PR suggested that the participant continued to use a rule that was correct previously, even when negative feedback was given.

In the current study, increased DC value in the middle frontal gyrus and lingual gyrus was negatively correlated with PR and PE values in cadets. In controls, the total accuracy was positively correlated with the DC value of the lingual gyrus. In addition, when the two groups combined into one group, there was a positive correlation between total accuracy and the DC value of the lingual gyrus and a negatively correlation between the PE value and the DC value of the lingual gyrus. These results suggested that the middle frontal gyrus and lingual gyrus involved in executive function. At the same time, it was important to note that these correlations were weak and should be used with caution.

Positron emission tomography, functional magnetic resonance imaging, and functional near-infrared spectroscopy studies have identified increased activation of the frontal area during the performance of the test (Berman et al., 1995; Fallgatter and Strik, 1998; Mentzel et al., 1998). Participants with prefrontal damage often performed worse on the card sorting test than those with non-frontal brain injury (Robinson et al., 1980; Arnett et al., 1994). Frontal patients always exhibited significantly more perseverative errors than controls, indicating that they failed to shift from one rule to another. The lingual gyrus is located on the medial aspect of the occipital lobe. It seems to be involved in visual processing for the orientation and direction of stimuli (Grill-Spector and Malach, 2004). Meanwhile, the lingual gyrus is also involved in visual memory (Zhang et al., 2016). Patients with lingual gyrus damage demonstrated impairments in visual learning (Bogousslavsky et al., 1987). Visual learning is an important part of the mental mechanism of card-sorting test. Thus, the increased centrality in the middle frontal gyrus and lingual gyrus with negatively correlation with PR and PE values in cadets in the current study may suggest that cadets exhibit better executive function. The differences were all located in the left hemisphere may reflecting the left lateralization of the language network.

The cadets in the Civil Aviation Flight University of China were college students; they had to complete all units and course requirements. In addition, they are flight cadets; during the 4 years at the university, they must also go through various flight training programs and earn their pilot’s licenses. Then, the cadets must finish their theoretical studies in the classroom in the first 2 years and complete their flight training in the flight simulator (50 h) and training plane (about 200 h) for the last 2 years. Meanwhile, undergraduates in other majors only need to complete the course requirements in 4 years. Therefore, the differences between the two groups were that the cadets were much busier than the controls, and that the cadets learned flight operations and the controls did not. Currently, a pilot’s training and work are complex cognitive tasks. Visual–spatial imagery, real-time monitoring, and decision making are the core factors of flying. These functions are mainly related to the prefrontal and occipital cortex (Stuss, 1992). Previous study found that the fighter pilots demonstrated superior cognitive control, and this superiority correlated with structural alterations in the white matter of the frontal (Roberts et al., 2010). Therefore, despite the relatively low level of centrality in middle frontal gyrus and lingual gyrus as freshmen, after the busy studying and flight training, the cadets improved their brain function. Meanwhile, the controls exhibited a downward trend during these years and the reason for this phenomenon needed more evidence. These different trends of the two groups eventually led to an interaction effect. In addition, in the current study, the enhanced DC value of the prefrontal cortex and occipital cortex and their negative correlation with the PR and PE values of the Berg Card-Sorting Test-64 in flight cadets might represent these differences. These results suggest that intensive learning and flight training might alter the functional pattern of the prefrontal and occipital cortices, which enhance cognitive function. These correlations were no longer significant after multiple correlations. The reason for these results might be the relatively small sample of participants. Therefore, a more definitive causal relationship would require more participants and more data.

This study had some limitations. First, cognitive assessments at the first visit were not performed. Thus, the differences between the time points of the two groups could not be compared. There was a possibility that the cadets exhibited significant lower executive function than controls when they were freshmen. But after training, the differences between the two groups disappeared. Second, we collected only resting-state fMRI data, and the explanation of our results could only be based on speculation. Therefore, task-based fMRI studies are required in the future. In addition, the findings of the current study should be confirmed using a larger sample size.

To summarize, this was the first study to assess the changes in brain activity pattern caused by flight training. The current study demonstrated increased functional properties in cadets after flight training in the left middle frontal and left lingual gyri. In addition, these enhancements were negatively correlated with the PR and PE values of the Berg Card-sorting Test-64. These results suggest that flight training might promote the DC value of the prefrontal and occipital cortices and, in turn, enhance their executive function.

## Data availability statement

The datasets presented in this study can be found in online repositories. The names of the repository/repositories and accession number(s) can be found at: https://pan.baidu.com/s/1C0dLdo4xv9IODWSwXNzAlQ?pwd=9tjz.

## Ethics statement

The studies involving human participants were reviewed and approved by the Ethics Committee of the University of Electronic Science and Technology of China. The patients/participants provided their written informed consent to participate in this study.

## Author contributions

XC, ZW, and YM designed the experiment, wrote the article, and gave final approval of the version to be published. HW, HJ, and YL collected and analyzed the data. KX, JY, and CL had made a substantial contribution to the analysis and interpretation of the data, they also revised the article critically. All authors contributed to the article and approved the submitted version.

## Funding

This study was supported by the National Nature Science Foundation of China (no. U2033217) and Foundation of General Program in Civil Aviation Flight University of China (J2020-003).

## Conflict of interest

The authors declare that the research was conducted in the absence of any commercial or financial relationships that could be construed as a potential conflict of interest.

## Publisher’s note

All claims expressed in this article are solely those of the authors and do not necessarily represent those of their affiliated organizations, or those of the publisher, the editors and the reviewers. Any product that may be evaluated in this article, or claim that may be made by its manufacturer, is not guaranteed or endorsed by the publisher.

## Footnotes

^1^ http://www.restfmri.net/forum/restplus
